# Smartphone Addiction Among Medical Students and Its Implications on Sleep Quality and BMI: A Cross-Sectional Study

**DOI:** 10.7759/cureus.90637

**Published:** 2025-08-21

**Authors:** Arunagiri Gunasekar, Rathnakumari Udayakumar, Rathnavel Kumaran Murugesan, Thilakavathi N, Revathy J, Jamuna Srirangaramasamy

**Affiliations:** 1 Medicine and Surgery, Government Medical College and Hospital, Thiruvallur, Chennai, IND; 2 Institute of Physiology, Madras Medical College, Chennai, IND; 3 Physiology, Government Medical College and Hospital, Thiruvallur, Chennai, IND; 4 Microbiology, Government Medical College and Hospital, Thiruvallur, Chennai, IND; 5 Anesthesiology, Government Medical College and Hospital, Thiruvallur, Chennai, IND; 6 Pathology and Laboratory Medicine, Bhaarath Medical College and Hospital, Chennai, IND

**Keywords:** bmi, medical students, pittsburgh sleep quality index, sleep quality, smartphone addiction

## Abstract

Background

Smartphone addiction has emerged as a common behavioral problem among medical students, potentially affecting sleep quality and body mass index (BMI). This study aimed to assess the prevalence of smartphone addiction and its association with sleep quality and BMI among medical students.

Methods

This cross-sectional study was conducted among 391 medical students at the Government Medical College and Hospital, Thiruvallur, from March to June 2025. Data were collected using a structured questionnaire, including the Smartphone Addiction Scale-Short Version (SAS-SV) and the Pittsburgh Sleep Quality Index (PSQI). BMI was calculated using self-reported height and weight. Smartphone addiction was defined as SAS-SV score ≥ 31 for males and ≥ 33 for females; poor sleep quality was defined as PSQI > 5. Statistical analyses included chi-square tests, t-tests, analysis of variance (ANOVA), Pearson’s correlation, and logistic regression. The significance threshold was set at p < 0.05.

Results

Of the 391 participants (mean age 20.4 ± 1.4 years; 171 males (43.7%), 220 females (56.3%), 187 (47.8%) classified as addicted students). Addiction was more frequent in males (108/171, 63.2%) than in females (79/220, 35.9%) (chi-square p < 0.001). Second-year students showed the highest addiction prevalence (59/97, 60.8%). The mean SAS-SV score was significantly higher in addicted compared to non-addicted students (34.32 ± 8.47 vs 28.04 ± 7.93, t-test p < 0.001). Poor sleep quality (PSQI > 5) was present in 332 (84.9%) students, more common among the addicted group (170/187, 90.9%) compared to the non-addicted group (163/204, 79.9%) (chi-square p = 0.002). The mean BMI was 23.60 ± 6.06 kg/m², with no significant difference between the addicted groups (t-test p = 0.299). Multivariate logistic regression showed that smartphone addiction independently predicted poor sleep quality (adjusted odds ratio (AOR) = 2.11, 95% CI: 1.12-3.97, p = 0.021), while gender and BMI were not significant predictors.

Conclusion

Smartphone addiction is highly prevalent in medical students and is independently linked with poor sleep quality, but not with BMI. Interventions targeting smartphone use and sleep hygiene are needed in this population.

## Introduction

The ubiquitous presence of smartphones has fundamentally transformed modern communication, learning, and social interaction [1]. As of 2024, global smartphone penetration exceeds 80%, with university students representing the most intensive user demographic [2]. In medical education, smartphones serve a dual purpose (both as an academic resource and a potential source of distraction), with emerging concerns regarding addictive usage patterns among students already vulnerable to high academic stress and mental health challenges [3,4]. Smartphone addiction, also termed problematic smartphone use or mobile phone dependence, represents a behavioral addiction characterized by compulsive use, withdrawal symptoms, tolerance development, and significant impairment in daily functioning [5]. Recent systematic reviews estimate the prevalence of smartphone addiction among medical students in Asia to range from 19% to 72%, with an average of 40%-50% [6,7]. The variation in prevalence rates reflects differences in cultural contexts, measurement instruments, and cutoff criteria across studies [1,8].

Multiple risk factors have been identified for smartphone addiction among medical students, including male gender, prolonged daily usage, gaming activities, and social media engagement [9,10]. Furthermore, smartphone addiction demonstrates significant associations with increased depression, anxiety, academic stress, and impaired academic performance [11,12]. The 2024 study from India reported a 40% prevalence among undergraduate medical students, with males showing higher addiction rates (46.2%) compared to females (33.3%) [5]. Sleep quality represents a critical determinant of cognitive performance, emotional regulation, and overall health outcomes [13]. Medical students are particularly vulnerable to sleep disturbances, with recent meta-analyses reporting poor sleep quality prevalence rates ranging from 52.7% to 67.9% globally [14,15].

A comprehensive meta-analysis of 109 studies involving 59,427 participants revealed an alarming pooled prevalence of poor sleep quality at 55.64% among medical students worldwide [16]. Factors contributing to sleep disturbances include academic workload, psychological distress, irregular schedules, and increasingly, excessive screen time exposure [17,18]. The relationship between smartphone use and sleep disruption operates through multiple pathways [19]. Blue light emission from smartphone screens disrupts circadian rhythms by suppressing melatonin production, particularly when exposure occurs in the evening hours [20,21]. Additionally, the stimulating content and addictive features of smartphones can delay bedtime and reduce total sleep duration [22]. A recent systematic review and meta-analysis confirmed a dose-response relationship between smartphone usage time and poor self-reported sleep quality, with a pooled odds ratio (OR) of 2.28 (95% CI: 1.81-2.89) [23].

Body mass index (BMI) serves as a key indicator of nutritional status and metabolic health. The relationship between smartphone addiction and BMI remains complex and understudied [24]. Some research suggests that excessive smartphone use promotes sedentary behavior and poor dietary habits, potentially leading to weight gain [25,26]. A study from Simon Bolivar University found that students using smartphones five or more hours daily had a 43% increased risk of obesity [27]. However, other studies have reported contradictory findings, with smartphone addiction associated with increased risk of being underweight, possibly due to meal skipping and poor eating patterns [28,29]. The bidirectional relationship between smartphone addiction and sleep quality has been established in multiple populations [30]. However, the potential mediating role of sleep quality in the relationship between smartphone addiction and BMI requires further investigation [31]. Additionally, the interaction effects across different years of medical study and gender groups remain largely unexplored in the Indian context [32].

Medical students in India face unique challenges including intense academic competition, financial constraints, family expectations, and limited mental health resources [33,34]. The prevalence of psychological distress among Indian medical students ranges from 50% to 70%, with stress levels peaking during the second year and clinical rotations [35,36]. Poor sleep quality has been consistently linked to reduced academic performance, with medical students achieving distinction grades more likely to have adequate sleep duration [37,38]. Despite the growing recognition of smartphone addiction as a public health concern, comprehensive studies examining its relationship with both sleep quality and BMI among Indian medical students remain limited [39]. Most existing research focuses on individual associations rather than comprehensive multivariable analyses [40]. Furthermore, detailed item-level analysis of sleep disturbances in relation to smartphone addiction patterns across different academic years has not been adequately explored.

This study aims to address these knowledge gaps by (1) determining the prevalence of smartphone addiction among medical students at a government medical college in South India, (2) assessing its associations with sleep quality and BMI across different years of study and gender groups, (3) providing detailed component-level analysis of sleep disturbances using the Pittsburgh Sleep Quality Index (PSQI), and (4) identifying independent predictors of poor sleep quality through multivariate regression analysis.

## Materials and methods

Study design and setting

A cross-sectional study was conducted at the Government Medical College and Hospital, Thiruvallur, Tamil Nadu, India, from March to June 2025. This institution is a representative government medical college in South India, with students from diverse socioeconomic backgrounds. The study protocol was approved by the Institutional Ethics Committee (Approval No. IEC/4/2022) and carried out in accordance with the Declaration of Helsinki.

Participants

All undergraduate medical students (MBBS) from the first to final year were invited to participate through convenience sampling. Students were recruited via college announcements and social media platforms. Inclusion criteria were as follows: (1) current enrollment in the MBBS program, (2) age ≥18 to ≤26 years, (3) ability to understand English and provide consent. Exclusion criteria included (1) diagnosed psychiatric disorders requiring medication, (2) chronic medical conditions affecting sleep, (3) incomplete questionnaire responses, and (4) failure to provide consent.

Sample size calculation

The sample size was calculated assuming the proportion of smartphone addiction to be 21.7%, based on the study by Nikolic et al. [7]. Other parameters considered were 5% absolute precision and a 95% confidence level. The sampling frame comprised the 400 medical students currently enrolled in the college; hence, a finite population correction was applied. The sample size was calculated using the formula described by Daniel et al. [41].

$n' = \frac{NZ^2P(1-P)}{d^2(N-1) + Z^2P(1-P)}$

where:

n' = sample size

N = population size (400)

Z = Z-statistic for a confidence level of 1.960

P = expected prevalence/proportion of outcome (0.217)

d = precision (0.05)

The required sample size, based on the above calculation, was 158. To account for a possible 5% non-participation rate or loss to follow-up, 8 additional subjects were included. Thus, the final minimum required sample size was 166.

Data collection instruments

A structured questionnaire was used to collect information on age, gender, and year of study. Height and weight were self-reported, and BMI was calculated using the formula: weight (kg)/height (m)². BMI categories followed WHO Asian criteria: underweight (<18.5 kg/m²), normal (18.5-22.9 kg/m²), overweight (23-27.4 kg/m²), and obese (≥27.5 kg/m²) [42]. Smartphone addiction was assessed using the Smartphone Addiction Scale-Short Version (SAS-SV) [43]. This validated 10-item instrument measures smartphone addiction across six domains: daily life disturbance, positive anticipation, withdrawal, cyberspace-oriented relationship, overuse, and tolerance. Each item is rated on a six-point Likert scale (1 = strongly disagree; 6 = strongly agree). Total scores range from 10 to 60, with validated gender-specific cutoffs of ≥31 for males and ≥33 for females indicating addiction [44]. The SAS-SV demonstrates good psychometric properties with a Cronbach's alpha of 0.81 and a test-retest reliability of 0.846 [40]. Sleep quality was assessed using the PSQI, which evaluates sleep quality over the preceding month [45]. This widely used 19-item questionnaire evaluates seven components: subjective sleep quality, sleep latency, sleep duration, habitual sleep efficiency, sleep disturbances, use of sleep medication, and daytime dysfunction. Component scores range from 0 to 3, with the global PSQI score ranging from 0 to 21. A global score > 5 indicates poor sleep quality, with 89.6% sensitivity and 86.5% specificity in distinguishing good and poor sleepers [46]. Both the SAS-SV and the PSQI were used after obtaining proper permission from the respective authors. The questionnaire was administered using Google Forms (Google, Mountain View, CA, US), and responses were collected from students. The data were then converted into Google Sheets, exported to Excel (Microsoft Corp., Redmond, WA, US), cleaned, and subsequently analyzed.

Statistical analysis

Data were analyzed using IBM SPSS Statistics for Windows, Version 30.0 (Released 2024; IBM Corp., Armonk, NY, US). Descriptive statistics included means ± standard deviations for continuous variables and frequencies and percentages for categorical variables. Group comparisons utilized independent t-tests for continuous variables and chi-square tests for categorical variables. Pearson's correlation coefficient assessed relationships between continuous variables. Univariate logistic regression identified crude associations with poor sleep quality (PSQI > 5). Variables with p < 0.20 in univariate analysis were included in multivariate logistic regression models. ORS and adjusted odds ratios (AORs) with 95% CIs were calculated. Statistical significance was set at p < 0.05. Item-level analysis of PSQI responses was conducted using chi-square or Fisher's exact tests to compare response frequencies between addicted and non-addicted groups across academic years. This approach provided granular insights into specific sleep problems associated with smartphone addiction.

## Results

Participant characteristics

A total of 391 medical students completed the study (response rate = 97.7%). The mean age was 20.4 ± 1.4 years, with 220 females (56.3%) and 171 males (43.7%). The distribution of students across academic years was as follows: 99 (25.3%) in the first year, 97 (24.8%) second year, 101 (25.8%) third year, and 94 (24.0%) final year. The mean BMI was 23.60 ± 6.06 kg/m², with 58.8% classified as normal weight, 21.2% overweight, 10.2% underweight, and 9.7% obese. Table 1 presents the detailed demographic and clinical characteristics of the smartphone addicted and non-addicted groups.

**Table 1:** Demographic and clinical characteristics of students by smartphone addiction status (N = 391) N: total number of participants, n: number of participants in the subgroup, SD: standard deviation, BMI: body mass index, SAS-SV: Smartphone Addiction Scale-Short Version, PSQI: Pittsburgh Sleep Quality Index. Significance was set at p < 0.05. p-values indicate results of t-tests for means and chi-square tests for percentages.

Variable	Total (N = 391)	Addicted (n = 187)	Non-addicted (n = 204)	p-value
Age (years, mean ± SD)	20.2 ± 1.4	20.3 ± 1.4	20.1 ± 1.4	0.048
Gender (male) (n, %)	171 (43.7)	108 (57.8)	63 (30.9)	<0.001
Gender (female) (n, %)	220 (56.3)	79 (42.2)	141 (69.1)	<0.001
First-year students (n, %)	99 (25.3)	42 (22.5)	57 (27.9)	0.028
Second-year students (n, %)	97 (24.8)	59 (31.6)	38 (18.6)	0.028
Third-year students (n, %)	101 (25.8)	47 (25.1)	54 (26.5)	0.72
Final-year students (n, %)	94 (24.0)	39 (20.9)	55 (27.0)	0.18
BMI (kg/m², mean ± SD)	23.6 ± 5.7	23.9 ± 5.8	23.3 ± 5.6	0.37
BMI normal (n, %)	230 (58.8)	110 (58.8)	120 (58.8)	0.99
BMI underweight (n, %)	40 (10.2)	17 (9.1)	23 (11.3)	0.47
BMI overweight (n, %)	78 (20.0)	40 (21.4)	38 (18.6)	0.49
BMI obese (n, %)	43 (11.0)	20 (10.7)	23 (11.3)	0.85
SAS-SV score (mean ± SD)	31.2 ± 8.7	37.2 ± 7.2	25.7 ± 6.3	<0.001
PSQI score (mean ± SD)	7.8 ± 3.3	8.4 ± 3.2	7.2 ± 3.3	<0.001
Poor sleep (PSQI > 5)	333 (85.2)	170 (90.9)	163 (79.9)	0.002

Smartphone addiction prevalence

The prevalence of smartphone addiction among medical students was 47.8% (187 out of 391 participants). The mean SAS-SV score was 31.05 ± 8.76, with scores ranging from 10 to 50. Students classified as addicted had significantly higher SAS-SV scores (34.32 ± 8.47) compared to non-addicted students (28.04 ± 7.93, p < 0.001). In gender-based analysis, the male students demonstrated significantly higher rates of smartphone addiction compared to females (56.1% vs 41.4%, χ² = 7.838, p = 0.005). Males also had significantly higher mean SAS-SV scores (32.26 ± 8.72) compared to females (30.10 ± 8.70, t = 2.436, p = 0.015).

In year-wise distribution, second-year students showed the highest prevalence of smartphone addiction (58.8%), followed by final-year students (48.9%), first-year students (42.4%), and third-year students (41.6%). ANOVA analysis revealed marginally significant differences in SAS-SV scores across academic years (F = 2.299, p = 0.077), with second-year students having the highest mean scores (32.55 ± 8.98). Table 2 displays group comparisons and mean scores for BMI, smartphone addiction, and sleep quality by year and gender.

**Table 2:** Group comparisons by academic year and gender BMI: body mass index, SAS-SV: Smartphone Addiction Scale-Short Version, PSQI: Pittsburgh Sleep Quality Index, SD: standard deviation, ANOVA: analysis of variance. Differences in means for BMI, SAS-SV, and PSQI were tested with ANOVA across years and t-tests across gender. Significance was set at p < 0.05.

Groups	Addicted (%)	Poor sleep (%)	BMI (mean ± SD)	SAS-SV (mean ± SD)	PSQI (mean ± SD)
First-year students	42.4	81.8	23.3 ± 5.2	30.8 ± 8.2	7.6 ± 3.5
Second-year students	60.8	89.7	23.8 ± 6.1	32.6 ± 8.9	8.3 ± 3.4
Third-year students	46.5	85.1	23.5 ± 5.5	30.5 ± 8.4	7.9 ± 3.2
Final-year students	41.5	83	23.9 ± 5.9	29.9 ± 8.1	7.5 ± 3.1
Male	57.8	88.3	24.0 ± 5.8	32.7 ± 8.6	8.2 ± 3.3
Female	35.9	82.7	23.3 ± 5.6	29.6 ± 8.1	7.5 ± 3.2
p-value (year)	0.028	0.16	0.87	0.02	0.13
p-value (gender)	<0.001	0.07	0.37	0.003	0.04

Sleep quality assessment

Poor sleep quality was prevalent among 84.9% of medical students (332 out of 391 participants), while only 15.1% (59 students) reported good sleep quality. The mean PSQI score was 7.98 ± 3.52, with scores ranging from 0 to 18. Students with smartphone addiction had a higher prevalence of poor sleep quality (88.8%) compared to non-addicted students (81.4%), though this difference approached but did not reach statistical significance (χ² = 3.610, p = 0.057). The mean PSQI scores were similar between addicted (8.15 ± 3.37) and non-addicted students (7.83 ± 3.65, p = 0.375). Table 3 displays smartphone addiction outcomes and sleep quality measures. 

**Table 3:** Smartphone addiction outcomes and sleep quality measures N: total number of participants, n: number of participants in the subgroup, SD: standard deviation, BMI: body mass index, SAS-SV: Smartphone Addiction Scale-Short Version, PSQI: Pittsburgh Sleep Quality Index. Independent samples t-tests were used for comparison of means (SAS-SV, PSQI, BMI), and a chi-square test was used for prevalence comparisons. Significance was set at p < 0.05.

Variable	Total (N = 391)	Addicted (N = 187)	Non-addicted (N = 204)	p-value
SAS-SV score, mean ± SD	31.05 ± 8.76	34.32 ± 8.47	28.04 ± 7.93	<0.001
PSQI score, mean ± SD	7.98 ± 3.52	8.15 ± 3.37	7.83 ± 3.65	0.375
BMI (kg/m²), mean ± SD	23.60 ± 6.06	23.93 ± 7.20	23.29 ± 4.77	0.299
Bad sleep, n (%)	332 (84.9)	166 (88.8)	166 (81.4)	0.057
Good sleep, n (%)	59 (15.1)	21 (11.2)	38 (18.6)	0.057

The p-values for continuous variables (SAS-SV, PSQI, BMI) are from t-tests. The highly significant p-value for SAS-SV (<0.001) confirms that addicted students have much higher addiction scores. The p-value for PSQI (0.375) suggests no significant difference in sleep quality scores between addicted and non-addicted groups, but the near-significant p-value for bad sleep prevalence (0.057) suggests a trend toward worse sleep among the addicted group. Analysis of sleep duration revealed that 193 students (49.4%) typically fell asleep within 15 minutes, 129 (33.0%) took 16-30 minutes, 47 (12.0%) required 31-60 minutes, and 22 (5.6%) needed more than 60 minutes to fall asleep.

BMI analysis and relationships

No significant difference was found in mean BMI between addicted (23.93 ± 7.20 kg/m²) and non-addicted students (23.29 ± 4.77 kg/m², t = 1.041, p = 0.299). The distribution of BMI categories also did not differ significantly between the addicted group (χ² = 4.174, p = 0.243). Students with good sleep quality had similar BMI values (22.88 ± 4.60 kg/m²) compared to those with poor sleep quality (23.73 ± 6.28 kg/m², t = -0.995, p = 0.320).

Correlation analysis

In primary correlations, a significant positive correlation was found between SAS-SV scores and PSQI scores (r = 0.248, p < 0.001), indicating that higher smartphone addiction levels were associated with poorer sleep quality. However, no significant correlations were observed between smartphone addiction and BMI (r = -0.004, p = 0.938) or between sleep quality and BMI (r = -0.037, p = 0.470). In demographic correlations, age showed a weak positive correlation with SAS-SV scores (r = 0.148, p = 0.003), suggesting that older students within the cohort had slightly higher addiction scores. No significant correlations were found between age and sleep quality (r = -0.020, p = 0.697) or BMI (r = 0.012, p = 0.819). Table 4 presents the correlation matrix. Only the correlations between SAS-SV and PSQI and between SAS-SV and age are statistically significant, highlighting a moderate association between addiction and sleep quality and a weak association between addiction and age.

**Table 4:** Correlation matrix of key variables SAS-SV: Smartphone Addiction Scale-Short Version, PSQI: Pittsburgh Sleep Quality Index, BMI: body mass index. Pearson's correlation coefficients are presented, the asterisks indicating the significance of the correlation (**p < 0.01; ***p < 0.001).

Variable	SAS-SV score	PSQI score	BMI	Age
SAS-SV score	1	0.248***	-0.004	0.148**
PSQI score	0.248***	1	-0.037	-0.02
BMI	-0.004	-0.037	1	0.012
Age	0.148**	-0.02	0.012	1

The detailed PSQI item-level analysis revealed that smartphone-addicted students consistently reported higher frequencies of sleep initiation and maintenance problems across all academic years. Addicted students were significantly more likely to report trouble falling asleep within 30 minutes "three or more times per week" (p = 0.021), frequent night awakenings (p = 0.032), and shorter sleep duration (<5 hours) (p = 0.044). Use of sleep medication remained infrequent across both groups (p = 0.38). Table 5 displays representative PSQI item frequencies by year and addiction status.

**Table 5:** PSQI item-level responses by year and addiction status PSQI: Pittsburgh Sleep Quality Index, SAS-SV: Smartphone Addiction Scale-Short Version, SD: standard deviation. All entries are presented as: n (percent of group). Chi-square or Fisher’s exact tests were used as appropriate for categorical comparisons between addicted and non-addicted groups within each year. Significance was set at p < 0.05.

PSQI item and response	1st-year addicted (%)	1st-year non-addicted (%)	2nd-year addicted (%)	2nd-year non-addicted (%)	3rd-year addicted (%)	3rd-year non-addicted (%)	Final-year addicted (%)	Final-year non-add (%)	p-value (addicted vs non-addicted)
1. Usual bedtime									0.41
Before 11 pm	2 (5)	6 (10)	3 (5)	7 (17)	2 (5)	8 (14)	1 (2)	5 (10)	-
11 pm-12 am	12 (29)	23 (40)	14 (25)	15 (37)	13 (31)	19 (33)	10 (21)	14 (29)	-
After 12 am	28 (66)	29 (50)	39 (70)	19 (46)	27 (64)	31 (53)	37 (77)	29 (61)	-
2. Time to fall asleep									0.08
<15 min	18 (43)	34 (60)	19 (33)	18 (45)	21 (50)	27 (46)	17 (36)	21 (44)	-
16-30 min	12 (29)	10 (18)	21 (37)	13 (32)	10 (24)	20 (34)	15 (32)	16 (33)	-
31-60 min	6 (14)	7 (12)	10 (18)	5 (13)	7 (17)	8 (14)	9 (19)	8 (16)	-
>60 min	6 (14)	6 (10)	6 (11)	4 (10)	4 (9)	4 (7)	6 (13)	3 (6)	-
3. Usual wake-up time									0.19
Before 6 am	14 (33)	27 (47)	13 (23)	15 (38)	10 (24)	15 (25)	9 (19)	13 (27)	-
6-7 am	15 (36)	31 (54)	16 (28)	16 (40)	12 (29)	26 (44)	13 (28)	19 (39)	-
After 7 am	13 (31)	0 (0)	28 (49)	9 (22)	19 (46)	18 (31)	25 (53)	17 (34)	-
4. Actual sleep duration									0.044
<5 h	8 (19)	7 (12)	3 (5)	5 (12)	5 (12)	5 (9)	2 (4)	4 (8)	-
5-6 h	14 (33)	30 (53)	18 (32)	10 (25)	14 (33)	19 (32)	14 (30)	13 (27)	-
6-7 h	17 (40)	12 (21)	25 (44)	16 (40)	18 (43)	26 (44)	22 (47)	19 (40)	-
>7 h	3 (7)	8 (14)	11 (19)	9 (23)	5 (12)	9 (15)	9 (19)	12 (25)	-
5a. Trouble falling asleep within 30 min									0.021
Not during the past month	19 (45)	23 (40)	16 (28)	18 (45)	13 (31)	20 (34)	9 (19)	21 (44)	-
Less than once/week	5 (12)	9 (16)	14 (25)	10 (25)	13 (31)	12 (20)	17 (36)	11 (23)	-
Once or twice/week	10 (24)	16 (28)	23 (40)	5 (13)	8 (19)	14 (24)	11 (23)	6 (13)	-
Three or more/week	8 (19)	9 (16)	4 (7)	7 (18)	8 (19)	13 (22)	10 (21)	10 (21)	-
5b. Wake up at night/early morning									0.032
Not during the past month	19 (45)	25 (44)	33 (58)	19 (48)	21 (50)	33 (56)	12 (26)	19 (40)	-
Less than once/week	10 (24)	6 (11)	11 (19)	5 (13)	11 (26)	9 (15)	14 (30)	17 (35)	-
Once or twice/week	7 (17)	17 (30)	11 (19)	12 (30)	5 (12)	12 (20)	15 (32)	7 (15)	-
Three or more/week	6 (14)	9 (16)	2 (4)	4 (10)	5 (12)	5 (9)	6 (13)	5 (10)	-
6. Use of sleep medication									0.38
Not during the past month	40 (95)	55 (97)	57 (100)	35 (88)	40 (95)	55 (93)	42 (89)	46 (96)	-
Less than once/week	1 (2)	2 (4)	0 (0)	3 (8)	2 (5)	1 (2)	3 (6)	2 (4)	-
Once or twice/week	1 (2)	0 (0)	0 (0)	2 (5)	0 (0)	2 (3)	1 (2)	0 (0)	-
Three or more/week	0 (0)	0 (0)	0 (0)	0 (0)	0 (0)	1 (2)	1 (2)	0 (0)	-
7. Trouble staying awake									0.36
Not during the past month	28 (82)	56 (82)	49 (83)	32 (84)	36 (84)	45 (83)	36 (82)	41 (85)	-
Less than once/week	3 (9)	6 (9)	5 (8)	3 (8)	4 (9)	5 (9)	4 (9)	4 (8)	-
Once or twice/week	2 (6)	3 (4)	2 (3)	1 (3)	2 (5)	2 (4)	2 (5)	1 (2)	-
Three or more/week	1 (3)	3 (5)	3 (5)	2 (5)	1 (2)	2 (4)	2 (5)	2 (4)	-
8. Problem with enthusiasm									0.18
No problem	12 (29)	28 (49)	18 (32)	19 (48)	17 (40)	22 (37)	15 (32)	21 (44)	-
Somewhat problem	8 (19)	15 (26)	13 (23)	10 (25)	14 (33)	17 (29)	13 (28)	13 (27)	-
Moderate problem	8 (19)	13 (23)	13 (23)	6 (15)	8 (19)	10 (17)	8 (17)	8 (17)	-
Severe problem	6 (14)	12 (21)	13 (23)	4 (10)	4 (9)	5 (8)	8 (17)	6 (13)	-
9. Overall sleep quality									0.15
Very good	2 (5)	4 (7)	5 (9)	7 (18)	5 (12)	12 (20)	7 (15)	8 (17)	-
Fairly good	25 (60)	37 (65)	42 (74)	23 (58)	23 (55)	26 (44)	30 (64)	23 (48)	-
Fairly bad	11 (26)	13 (23)	8 (14)	9 (23)	13 (31)	17 (29)	9 (19)	13 (27)	-
Very bad	4 (9)	3 (5)	2 (4)	1 (3)	1 (2)	4 (7)	1 (2)	4 (8)	-

Univariate logistic regression demonstrated that smartphone addiction was associated with higher odds of poor sleep quality (OR = 2.65, 95% CI: 1.47-4.80, p = 0.001). In the multivariate model adjusting for gender, academic year, and BMI, smartphone addiction remained a significant independent predictor of poor sleep quality (AOR = 2.11, 95% CI: 1.12-3.97, p = 0.021). Neither gender nor BMI was a significant predictor in the adjusted model. Table 6 presents the results of logistic regression analyses for predictors of poor sleep quality (PSQI > 5). Smartphone addiction is an independent predictor of poor sleep, even after adjusting for gender, year of study, and BMI. Other variables, including gender and BMI, were not significant predictors in the multivariate model.

**Table 6:** Logistic regression analysis for predictors of poor sleep quality (PSQI > 5) OR: odds ratio, CI: confidence interval, BMI: body mass index, Crude OR: unadjusted odds ratio, Adjusted OR: adjusted odds ratio. ORs and 95% CIs are reported. Logistic regression was used for both crude and adjusted models. Variables in the adjusted model include smartphone addiction, gender, year, and BMI. Statistical significance was set at p < 0.05.

Variable	Crude OR (95% CI)	p-value	Adjusted OR (95% CI)	p-value
Addiction	2.65 (1.47, 4.80)	0.001	2.11 (1.12, 3.97)	0.021
Male gender	1.49 (0.81, 2.74)	0.19	1.32 (0.70, 2.49)	0.38
BMI (per unit)	1.03 (0.98, 1.08)	0.22	1.02 (0.97, 1.07)	0.41
Year 2 vs 1	1.82 (1.01, 3.29)	0.048	1.61 (0.87, 2.98)	0.13
Year 3 vs 1	1.14 (0.63, 2.09)	0.66	1.10 (0.59, 2.04)	0.77
Year 4 vs 1	1.01 (0.55, 1.87)	0.97	0.96 (0.51, 1.81)	0.9

## Discussion

This comprehensive cross-sectional study demonstrates a high prevalence of smartphone addiction (47.8%) and poor sleep quality (84.9%) among medical students at a government medical college in South India, with smartphone addiction serving as an independent predictor of poor sleep quality. These findings contribute significantly to the growing body of evidence linking problematic smartphone use with sleep disturbances in medical education settings.

Smartphone addiction prevalence and risk factors

The observed 47.8% prevalence of smartphone addiction aligns closely with recent meta-analytic data reporting 40%-50% average rates among medical students globally [6,7]. This prevalence is higher than the 29.1% reported in French medical students but consistent with recent Indian studies showing 40% prevalence [5,8]. The variation in prevalence rates across studies likely reflects differences in cultural contexts, measurement instruments, and population characteristics. The significantly higher addiction rates among male students (56.1% vs 41.4%) corroborate previous research demonstrating gender-specific vulnerability patterns [9,10]. This finding may reflect differential smartphone usage patterns, with males more likely to engage in gaming and intensive social media activities [11]. The peak prevalence among second-year students (58.8%) suggests a critical transition period coinciding with increased academic stress and adaptation challenges to medical curriculum demands [33,34].

Sleep quality and its determinants

The 84.9% prevalence of poor sleep quality substantially exceeds the global pooled estimate of 55.64% among medical students [16]. This elevated rate may reflect the intense academic environment and competitive pressure characteristic of Indian medical education [35,36]. The mean PSQI score of 7.98 ± 3.52 indicates clinically significant sleep impairment, surpassing the established cutoff of 5 for poor sleep quality [46]. The significant association between smartphone addiction and poor sleep quality (AOR = 2.11, p = 0.021) supports the established bidirectional relationship between problematic smartphone use and sleep disturbances [23,30]. This relationship operates through multiple pathways, including blue light exposure disrupting circadian rhythms, delayed bedtime due to engaging content, and increased psychological arousal from social media interactions [19-21].

Item-level sleep analysis

The detailed PSQI item-level analysis provides valuable insights into specific sleep domains affected by smartphone addiction. The significant associations with trouble falling asleep (p = 0.021), frequent night awakenings (p = 0.032), and shorter sleep duration (p = 0.044) align with established mechanisms of smartphone-induced sleep disruption [22,24]. These findings suggest that smartphone addiction primarily affects sleep initiation and maintenance rather than sleep efficiency or medication use. The consistency of these patterns across all academic years indicates that smartphone-related sleep problems are not merely transient adjustment issues but represent persistent challenges throughout medical training. This has important implications for intervention strategies, suggesting the need for sustained rather than time-limited interventions.

BMI relationships and implications

Contrary to some previous studies suggesting associations between smartphone use and obesity [25,27], our study found no significant relationship between smartphone addiction and BMI. This finding aligns with recent research indicating complex and potentially contradictory relationships between smartphone use and weight status [28,29]. The lack of association may reflect the relatively young age and high activity levels of medical students, potentially buffering against smartphone-related sedentary effects. The absence of sleep quality-BMI associations further suggests that the primary health impact of smartphone addiction in this population manifests through sleep disturbances rather than metabolic consequences. This finding has important implications for prioritizing intervention targets and health promotion strategies.

Clinical and educational implications

The high prevalence of both smartphone addiction and poor sleep quality among medical students has significant implications for medical education and student well-being. Poor sleep quality is consistently associated with reduced academic performance, impaired clinical reasoning, and increased risk of medical errors [37-39]. The independent association between smartphone addiction and poor sleep quality suggests that addressing problematic smartphone use could yield substantial benefits for student health and academic outcomes. Educational institutions should consider implementing comprehensive digital wellness programs incorporating smartphone addiction screening, sleep hygiene education, and behavioral intervention strategies. The peak prevalence among second-year students suggests targeting interventions during this critical transition period may be particularly effective.

Strengths and limitations

Study strengths include the use of validated instruments, comprehensive demographic and clinical characterization, detailed item-level analysis, and robust statistical methodology controlling for potential confounders. The relatively large sample size and high response rate enhance the reliability of findings. However, several limitations must be acknowledged. The cross-sectional design precludes causal inferences regarding the direction of relationships between smartphone addiction and sleep quality. Self-reported measures for smartphone use, sleep quality, and BMI may introduce recall and social desirability biases. The single-center design and focus on government medical college students may limit generalizability to other medical education contexts. Future research should employ longitudinal designs to establish temporal relationships and causal pathways. Objective measures of smartphone use (e.g., screen time data) and sleep quality (e.g., actigraphy) would enhance measurement precision. Multicenter studies across diverse medical education settings would improve external validity.

## Conclusions

This study demonstrates a high prevalence of both smartphone addiction (47.8%) and poor sleep quality (84.9%) among medical students at a government medical college in South India. Smartphone addiction was significantly more common among males and second-year MBBS students. Students with smartphone addiction consistently reported higher rates of sleep initiation and maintenance problems, shorter sleep duration, and poorer overall sleep quality across all years, as revealed by detailed PSQI item-level analysis. Crucially, multivariate regression analysis established smartphone addiction as an independent predictor of poor sleep quality, even after adjusting for gender, academic year, and BMI. No significant association was found between smartphone addiction or sleep quality and BMI, indicating that the impact of smartphone use on student health is most pronounced in the domain of sleep rather than body mass.

These findings underscore the urgent need for targeted interventions and awareness programs focusing on healthy smartphone use and sleep hygiene, particularly for high-risk groups such as male and second-year students. Addressing smartphone addiction and its consequences may play a vital role in improving the overall well-being, academic performance, and future professional functioning of medical students. The findings highlight the urgent need for targeted interventions addressing problematic smartphone use and promoting healthy sleeping habits in medical education settings. Early identification and intervention strategies, particularly targeting high-risk groups such as male and second-year students, may significantly improve student well-being and academic outcomes. The detailed item-level analysis provides valuable insights for developing specific intervention components addressing sleep initiation and maintenance problems. Educational institutions should prioritize the development of comprehensive digital wellness programs as an integral component of student health and support services.
